# Supplementary material for: Multi-level quantum noise spectroscopy
Source: Nat Commun. 2021 Feb 11;12:967. doi: 10.1038/s41467-021-21098-3 (PMC7878521; doi:10.1038/s41467-021-21098-3)
Supplement: Supplementary file 1 — Supplementary Information [file 41467_2021_21098_MOESM1_ESM.pdf]

Supplementary Information: Multi-level Quantum Noise Spectroscopy

Youngkyu Sung[1,2], Antti Vepsäläinen[1], Jochen Braumüller[1], Fei Yan*[1], Joel I-Jan Wang[1], Morten Kjaergaard[1], Roni Winik[1], Philip Krantz†[1], Andreas Bengtsson‡[1], Philip Krantz[1], Alexander J. Melville[3], Bethany M. Niedzielski[3], Mollie E. Schwartz[3], David K. Kim[3], Jonilyn L. Yoder[3], Terry P. Orlando[1,2], Simon Gustavsson[1], Lorenza Viola[3], and William D. Oliver[1,2,3,4]

[1]Research Laboratory of Electronics, Massachusetts Institute of Technology, Cambridge, MA 02139, USA
[2]Department of Electrical Engineering and Computer Science, Massachusetts Institute of Technology, Cambridge, MA 02139, USA
[3]MIT Lincoln Laboratory, 244 Wood Street, Lexington, MA 02421, USA
[4]Department of Physics, Massachusetts Institute of Technology, Cambridge, MA 02139, USA

Contents

Supplementary Note 1 Device parameters 2

Supplementary Note 2 Measurement Setup 2

Supplementary Note 3 Pump-probe spectroscopy 3

Supplementary Note 4 Derivation of the Effective Hamiltonian describing the j-th spin-locking noise spectrometer 7

Supplementary Note 5 Derivation of Reduced Master Equation 9

Supplementary Note 6 Contributions of T_1 decay to the spin relaxation $\Gamma_{1\rho}$ 11

Supplementary Note 7 Separating out the contributions of T_1 decay and native dephasing 12

Supplementary Note 8 Advantages of the spin-locking QNS over the dynamic decoupling QNS 13

*Current address: Southern University of Science and Technology, Shenzhen, Guangdong, China 518055.
†Current address: Wallenberg Centre for Quantum Technology (WACQT), Chalmers University of Technology, Gothenburg, SE-41296, Sweden.
‡Current address: Microtechnology and Nanoscience, Chalmers University of Technology, Gothenburg, SE-41296, Sweden.

Supplementary Note 1 Device parameters

In the experiments, we tune the transmon to an operating point where its frequency is highly sensitive to flux noise, see Fig. 2(d) and Supplementary Figure 1. In addition to the sensor, there are two other transmon qubits in the same chip which are not used in the current experiment. At the operating point, the nearest-neighboring (N.N.) transmon is far detuned from the transmon sensor, such that it can be neglected, while the sensor operates (qubit frequency detuning $\Delta_{\text{N.N.}}/2\pi \equiv (\omega^{(0,1)}_{\text{N.N.}} - \omega^{(0,1)}_{\text{s}})/2\pi$ = 1.6 GHz, effective coupling strength $g_{\text{N.N.}}/2\pi \approx$ 15 MHz). Similarly, the next-nearest-neighboring (N.N.N.) transmon is also effectively isolated from the sensor, while it operates (qubit frequency detuning $\Delta_{\text{N.N.N.}}/2\pi \equiv (\omega^{(0,1)}_{\text{N.N.N.}} - \omega^{(0,1)}_{\text{s}})/2\pi$ = 230 MHz, effective coupling strength $g_{\text{N.N.N.}}/2\pi \approx$ 1.4 MHz). The device parameters are summarized in Supplementary Table 1.

Supplementary Table 1. Device parameters.

Parameter	Value
Total Josephson energy of the two junctions $E_{\text{J},\Sigma}$	11.16 (GHz)
Junction asymmetry d	0.0
Capacitive energy E_{c}	181.5 (MHz)
Flux bias at the operating point Φ_{ext}	0.170 (Φ_0)
$\|0\rangle$-$\|1\rangle$ transition frequency at the operating point $\omega^{(0,1)}_{\text{s}}/2\pi$	3.5435 (GHz)
Relaxation time T_1 for the $\|0\rangle$-$\|1\rangle$ transition $1/\Gamma^{(0,1)}_1$	58 (µs)
$\|1\rangle$-$\|2\rangle$ transition frequency at the operating point $\omega^{(1,2)}_{\text{s}}/2\pi$	3.3362 (GHz)
Relaxation time T_1 for the $\|1\rangle$-$\|2\rangle$ transition $1/\Gamma^{(1,2)}_1$	31 (µs)
Readout resonator frequency $\omega_{\text{r}}/2\pi$	7.249 (GHz)
[1] Linewidth of the readout resonator $\kappa_r/2\pi$	4.18 (MHz)
Effective dispersive coupling strength for the $\|0\rangle$-$\|1\rangle$ transition, $\chi^{(0,1)}/2\pi$	115 (kHz)
Effective dispersive coupling strength for the $\|1\rangle$-$\|2\rangle$ transition, $\chi^{(1,2)}/2\pi$	146 (kHz)

Supplementary Note 2 Measurement Setup

2.1 Cryogenic setup

The experiments were performed in a Leiden CF-450 dilution refrigerator with a base temperature of $20\,\text{mK}$. The device was magnetically shielded with a superconducting can surrounded by a Cryoperm-10 cylinder. There are two lines for input and output; we apply microwave readout tone and measuring the transmission of sample. All attenuators in the cryogenic samples are made by XMA and installed to remove excess thermal photons from higher-temperature stages. We pump the Josephson travelling wave parametric amplifier (JTWPA) to pre-amplify the readout signal at base tempearture [S7]. To avoid any back-action of the pump-signal from TWPA, we added a microwave isolator between the samples and the TWPA. On the RF output line, there is a high-electron mobility transistor (HEMT) amplifier (Cryo-1-12 SN508D) thermally connected to the $3\,\text{K}$ stage. Two microwave isolators allow for the signal to pass through to the amplifier without being attenuated, while taking all the reflected noise off of the amplifier and dumping it in a $50\,\Omega$ termination instead of reaching the sample.

2.2 Room temperature control

Outside of the cryostat, we have all of the control electronics which allow us to apply microwave signals used for the readout and control of the transmon sensor.

All the signals are added using microwave power splitters (Marki PD0R413) used in reverse. Pulse envelopes of qubit control signals and readout signals are programmed in Labber software and then uploaded to arbitrary waveform generators (AWG Keysight M3202A). Subsequently, the pulses generated by AWGs are mixed with coherent tone from RF sources (Rohde and Schwarz SGS100A). All components for generating signals are frequency-locked using the $10\,\text{MHz}$ reference clock in the Keysight PXIe Chassis M9019A. A detailed schematic is given in Fig S2.

2.3 Generation of engineered flux noise

We generate engineered flux noise waveforms using the method described in Supplementary Material 3 of Ref. [S5]. In all experiments presented in the main text, we consider the Lorentzian-shaped flux-noise PSD as follows:

$$S_\Phi(\omega) = \frac{P_0}{2\pi\omega_c}\left(\frac{1}{1+[(\omega-\omega_0)/\omega_c]^2} + \frac{1}{1+[(\omega+\omega_0)/\omega_c]^2}\right), \tag{1}$$

where P_0 denotes the noise power, $\omega_0/2\pi = f_0$ denotes the center frequency of the noise, and $\omega_c/2\pi = 2\,\text{MHz}$ denotes the half-width at half-maximum (HWHM) of the Lorentzian curve. As described in [S5], we discretely sample the noise spectrum by taking harmonics separated by the fundamental frequency, $4\,\text{kHz}$. The noise spectrum is sampled with a high-frequency cutoff

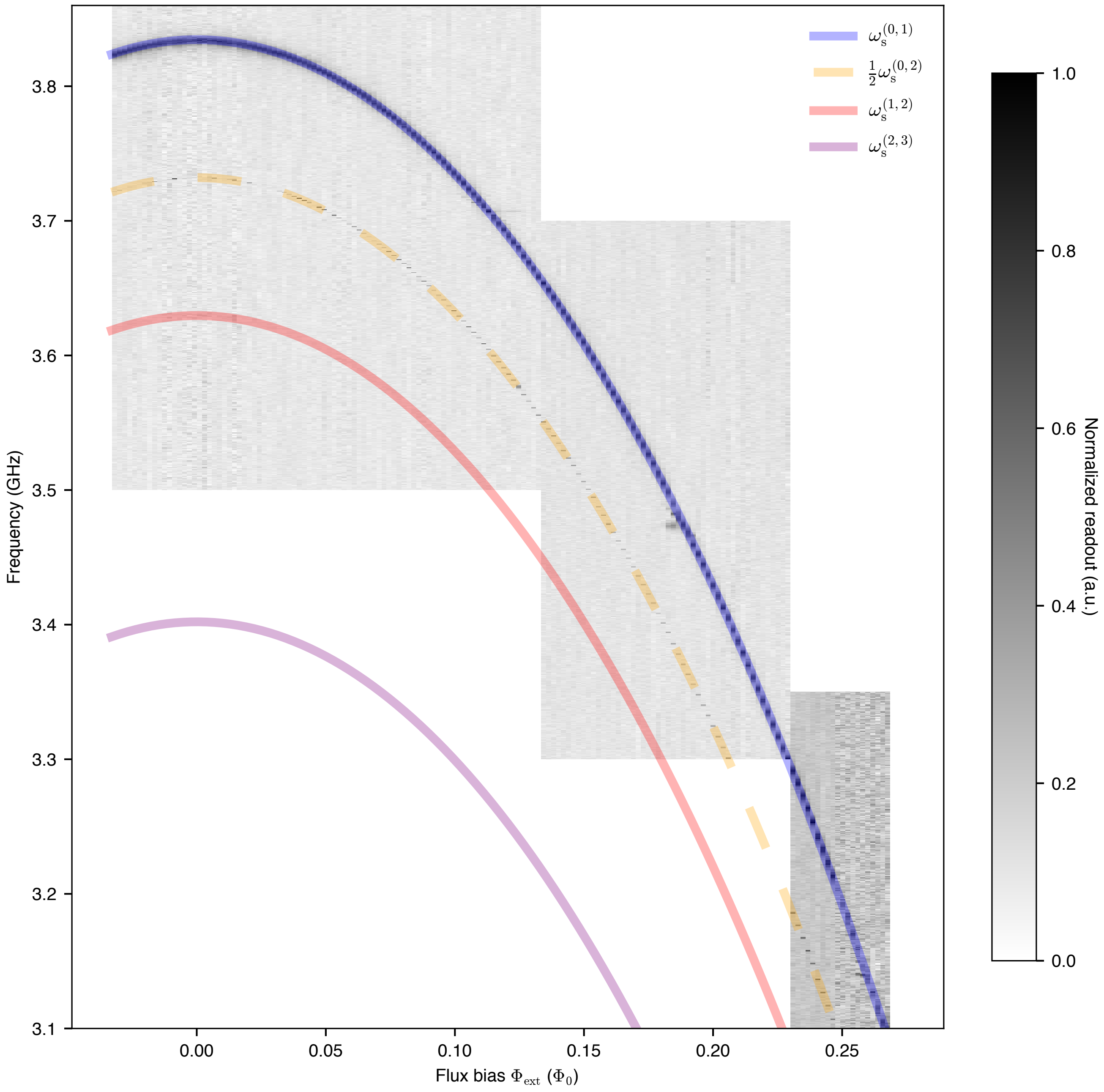

Supplementary Figure 1. Qubit spectroscopy as a function of the spectroscopy tone frequency and external magnetic flux threading the SQUID loop of the spectrometer. Solid and dashed curves are obtained by solving the eigen-energies of the circuit Hamiltonian based on the parameters summarized in Table. 1. Note that the dashed orange curve corresponds to the two-photon ($|0\rangle$–$|2\rangle$) transition frequency.

($\omega_0/2\pi + 50\,\mathrm{MHz}$) and a low-frequency cutoff $\max(0, (\omega_0/2\pi - 50\,\mathrm{MHz}))$. In each measurement, a new waveform is produced by an arbitrary waveform generator (AWG) and the total number of noise samples is 1,000. Each noise waveform has a duration of $100\,\mu\mathrm{s}$.

Supplementary Note 3 Pump-probe spectroscopy

In Fig. 3(a), we discussed the effect of multi-level dressing (the frequency shift, $\Omega - \lambda_j A_{\mathrm{drive}}$) by describing the deviation of measured Rabi frequency from the one expected in the two-level approximation. Here, we present experimental results of the pump-probe spectroscopy, which is an alternative approach to capture the effect of multi-level dressing.

Before describing the experimental results, we first present the dressed state picture for a driven multi-level system. We consider a multi-level transmon driven by an electromagnetic field, which is tuned to the frequency of the transmon's $|0\rangle$-$|1\rangle$ transition (Fig. S1). Then, the transmon-photon system can be written as follows:

$$H(t) = H_0 + H_{\mathrm{int}}(t), \tag{2}$$

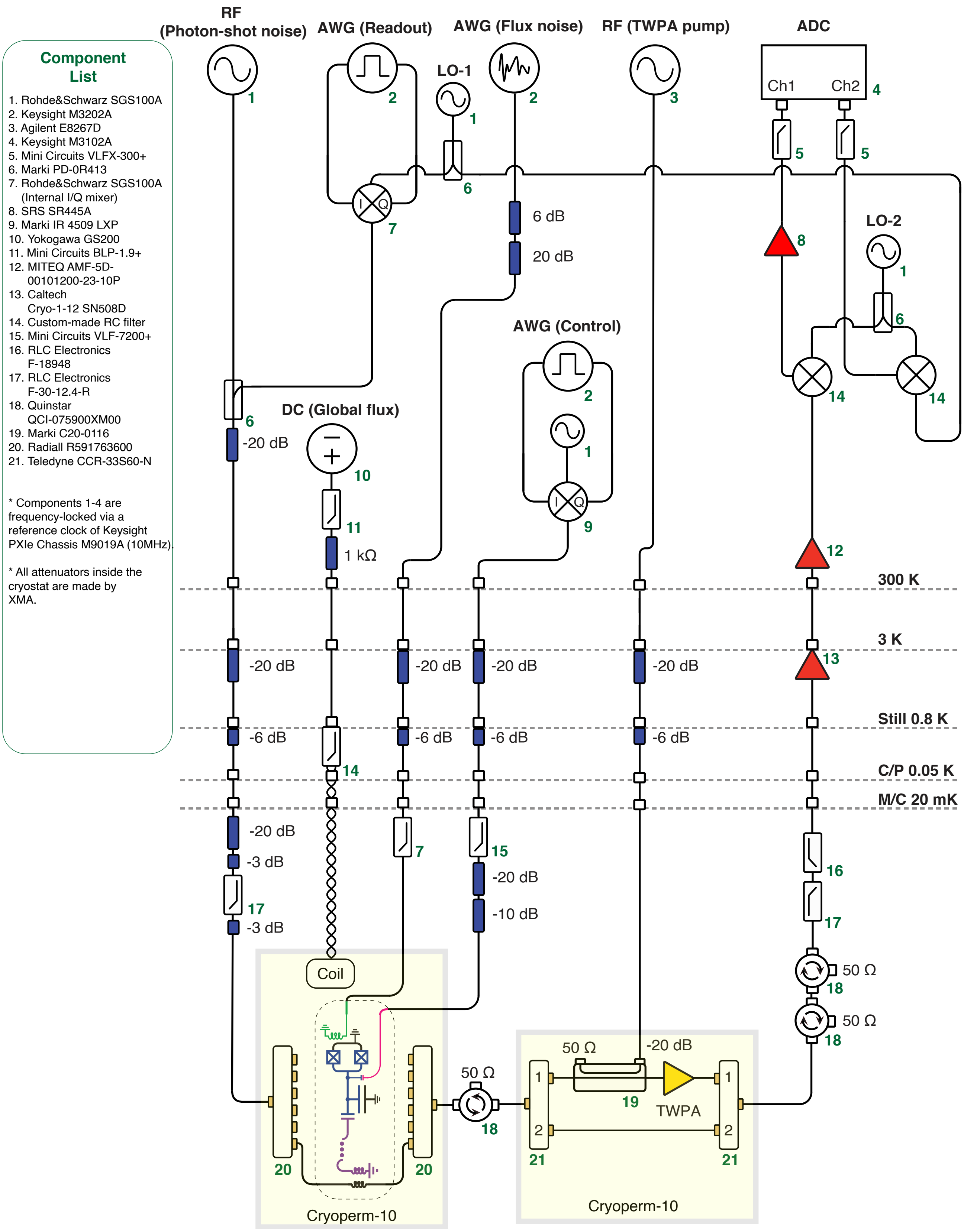

Supplementary Figure 2. Electronics and control wiring

where a time-independent Hamiltonian H_0 represents the sum of the energies of the transmon and the quantized mode of electromagnetic field (photon), and $H_{\rm int}(t)$ denotes the Hamiltonian describing the interaction between the transmon and the photon. We first choose a set of eigenstates of the interaction-free Hamiltonian H_0 as a basis. This basis corresponds to a tensor-product of the transmon states and photon states, $|j,n\rangle$ (Fig. S3). In this basis, the transmon-photon system can be represented as multiple ladders of quantized energy levels. Now, we introduce the interaction between the transmon and photon (red double-headed arrow in Fig. S4). In the case of a driven four-level transmon as illustrated in Fig. S4, the four transmon-photon product states $|0,n\rangle, |1,n-1\rangle, |2,n-2\rangle$, and $|3,n-3\rangle$ can be grouped as "the n-excitation manifold" (grey-filled), each having the same number n of excitations in total. The interaction between these product states leads to the formation of dressed states

$|+_n\rangle, |-_n\rangle, |2'_n\rangle$, and $|3'_n\rangle$; the dressed states correspond to the eigenstates of the Hamiltonian including the interaction. Note that single-photon transitions are allowed between the nearest-neighboring manifolds. Two-photon transitions are available between the next-nearest-neighboring manifolds.

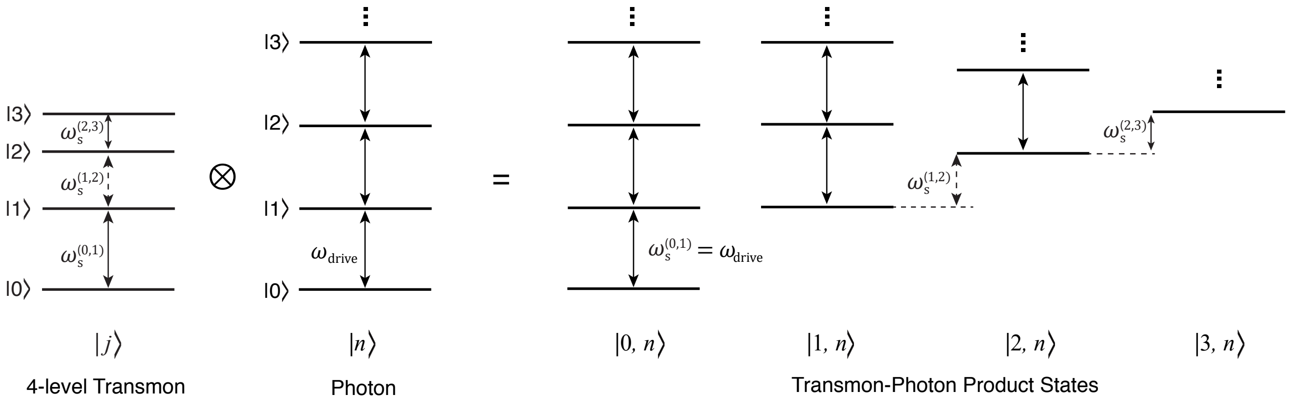

Supplementary Figure 3. Representation of (four-level-) transmon-photon product states. j denotes the excitation level of the transmon and n denotes the number of photons.

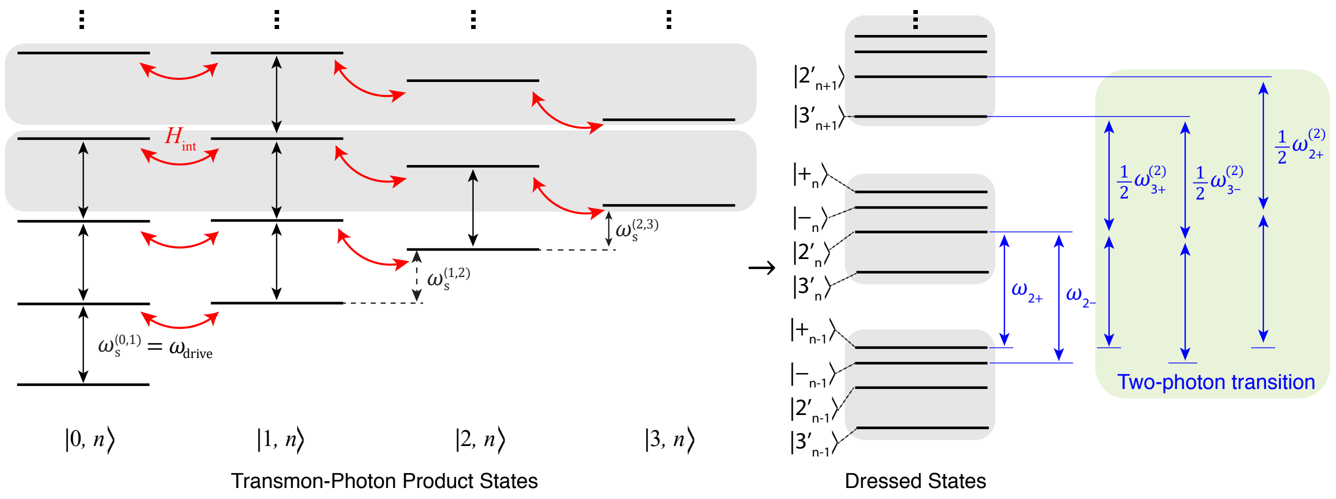

Supplementary Figure 4. Dressed state representation of the transmon-photon coupled system. The four product states $|0, n\rangle, |1, n-1\rangle, |2, n-2\rangle$, and $|3, n-3\rangle$ (grey filled) can be grouped as the n-excitation manifold. The interaction between the transmon and photon (red double-headed arrow, H_{int}) leads to the formation of dressed states. Note that single-photon transitions are allowed between the nearest-neighboring manifolds. Two-photon transitions are available between the next-nearest-neighboring manifolds.

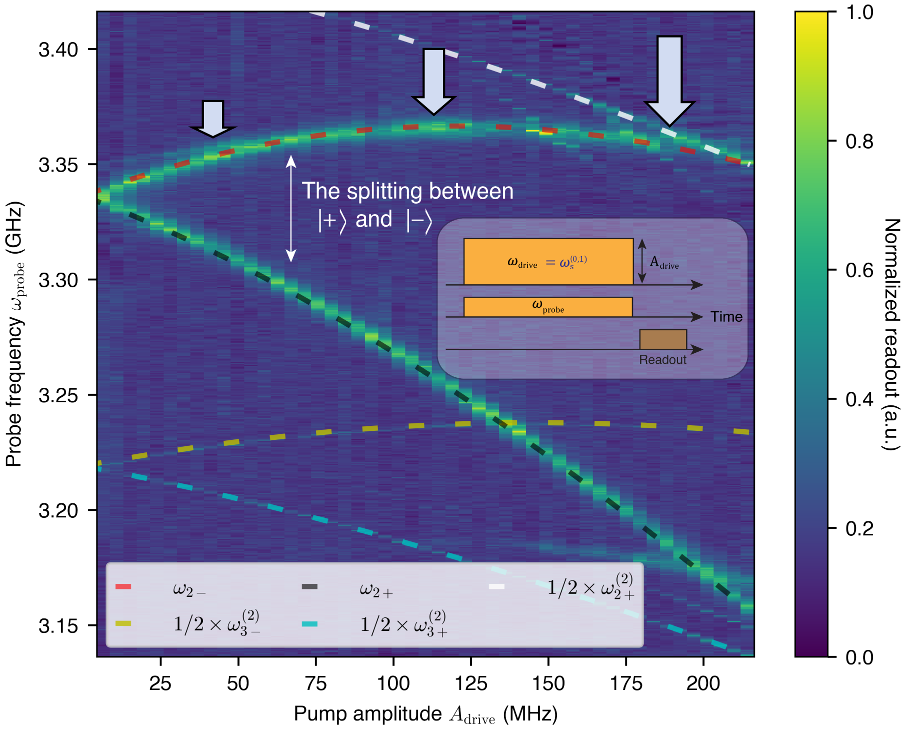

Supplementary Figure 5. Measurement of pump-probe spectroscopy. A strong pump tone is applied to the transmon sensor at the frequency of $|0\rangle$–$|1\rangle$ transition, $\omega_s^{(0,1)}$, which dresses the transmon sensor. We measure single- and two-photon transition frequencies between the dressed states as a function of the pump amplitude (A_{drive}) by measuring the absorption of a weak probe tone (ω_{probe}). The splitting between the dressed states $|+\rangle$ and $|-\rangle$ corresponds to the Vacuum Rabi splitting and is effectively pushed due the multi-level dressing effect. This effect becomes more significant as the pump amplitude A_{drive} increases. Dotted lines correspond to the simulation data based on the circuit parameters (Table 1).

In the pump-probe spectroscopy measurements, we drive the multi-level system with a strong pump tone at the frequency resonant with $|0\rangle$-$|1\rangle$ transition, which dresses the multi-level system. Then, we measure the level transitions between the dressed states by measuring the response of the system (absorption) to a weak probe tone. Fig. S5 shows the measurement of transition frequencies as a function of the pump amplitude $A_{\rm drive}$ and compares with the simulation data. Note that the splitting between the dressed states $|+\rangle$ and $|-\rangle$ corresponds to the vacuum Rabi splitting Ω. The vacuum Rabi splitting scales linearly with the drive amplitude (= pump amplitude) in the two-level approximation. However, in the case of a multi-level system, the splitting is effectively pushed due to the presence of higher transmon levels. The discrepancy between the vacuum Rabi splitting and the pump amplitude ($\Omega^{(j-1,j)} - A_{\rm drive}$) corresponds to the frequency shift due to the multi-level dressing, which is also measured in Fig. 3(a).

Supplementary Note 4 Derivation of the Effective Hamiltonian describing the j-th spin-locking noise spectrometer

As discussed in the main text, we consider an externally-driven d-level quantum sensor evolving under a noisy environment, which induces pure dephasing noise into the sensor. The Hamiltonian of the whole system $H(t)$ can be written as

$$H(t) = H_{\rm S}(t) + H_{\rm SB} + H_{\rm B}, \tag{3}$$

where $H_{\rm S}(t)$ denotes the time-dependent Hamiltonian of the driven multi-level sensor, $H_{\rm B}$ denotes the bath Hamiltonian, and $H_{\rm SB}$ denotes the sensor-bath interaction. Since we consider only pure-dephasing (σ_z-type) noise, the sensor-bath interaction can be written as

$$H_{\rm SB} = \hbar \sum_{j=1}^{d-1} B^{(j)} |j\rangle\langle j|, \tag{4}$$

where $|j\rangle\langle j|$ is the projector for j-th level of the multi-level sensor and $B^{(j)}$ is the bath operator that longitudinally couples to the j-th level of the sensor [S10]. Following the same notations used in the main text for Eq. (1), the Hamiltonian of the driven multi-level sensor is given by

$$H_{\rm S}(t) =\hbar \sum_{j=1}^{d-1} \Big[\omega_{\rm s}^{(j)} |j\rangle\langle j| + \lambda^{(j-1,j)} A_{\rm drive} \cos(\omega_{\rm drive} t)(\sigma_+^{(j-1,j)} + \sigma_-^{(j-1,j)})\Big]. \tag{5}$$

To reiterate, the sensor eigenenergies are $\hbar\omega_{\rm s}^{(j)}$ with the ground state energy set to zero. The raising and lowering operators of the sensor are denoted by $\sigma_+^{(j-1,j)} \equiv |j\rangle\langle j-1|$ and $\sigma_-^{(j-1,j)} \equiv |j-1\rangle\langle j|$, respectively. Here, we continuously drive the sensor with a continuous signal $A_{\rm drive}\cos(\omega_{\rm drive} t)$, where $A_{\rm drive}$ and $\omega_{\rm drive}$ correspond to the amplitude and the frequency of the driving field, respectively. The parameter $\lambda^{(j-1,j)}$ represents the strength of the $|j-1\rangle$–$|j\rangle$ transition relative to the $|0\rangle$–$|1\rangle$ transition with $\lambda^{(0,1)} \equiv 1$.

Now we move to the interaction picture with respect to the bath Hamiltonian $H_{\rm B}$. By introducing the time-dependent noise operator $B^{(j)}(t) \equiv e^{iH_{\rm B}t/\hbar} B^{(j)} e^{-iH_{\rm B}t/\hbar}$, the sensor-bath joint Hamiltonian can be written as (same as Eq. (1) in the main text)

$$\tilde{H}(t) =\hbar \sum_{j=1}^{d-1} \Big[\Big(\omega_{\rm s}^{(j)} + B^{(j)}(t)\Big) |j\rangle\langle j| + \lambda^{(j-1,j)} A_{\rm drive} \cos(\omega_{\rm drive} t)(\sigma_+^{(j-1,j)} + \sigma_-^{(j-1,j)})\Big], \tag{6}$$

Then, we move to the rotating frame, which rotates at the drive frequency $\omega_{\rm drive}$ with respect to the longitudinal axis of the sensor. To move to the rotating frame, we define the unitary operator $U_{\rm R}(t)$ as

$$U_{\rm R}(t) \equiv \sum_{j=1}^{d-1} \exp\left(-ij\omega_{\rm drive} t\right) |j\rangle\langle j|, \tag{7}$$

which determines the transformed Hamiltonian as follows:

$$\tilde{H}_{\rm R}(t) = U_{\rm R}^\dagger(t) \tilde{H}(t) U_{\rm R}(t) + i\dot{U}_{\rm R}^\dagger(t) U_{\rm R}(t). \tag{8}$$

Assuming that $\omega_{\rm drive}$ is larger then any other rate or frequency in this frame, we can perform the rotating wave approximation

(RWA), which leads to the Hamiltonian [S7]

$$
\begin{aligned}
\tilde{H}_{\rm RWA}(t) &= \hbar\sum_{j=1}^{d-1}\left[\left[\left(\omega_{\rm s}^{(j)} - j\omega_{\rm drive}\right) + B^{(j)}(t)\right]|j\rangle\langle j| + \lambda^{(j-1,j)}\left[\frac{A_{\rm drive}}{2}(\sigma_+^{(j-1,j)} + \sigma_-^{(j-1,j)})\right]\right] \\
&= \hbar\left[\begin{pmatrix} 0 & A_{\rm drive}/2 & 0 & \cdots & 0 \\ A_{\rm drive}/2 & \omega_{\rm s}^{(1)} - \omega_{\rm drive} & \lambda^{(1,2)}A_{\rm drive}/2 & \cdots & 0 \\ 0 & \lambda^{(1,2)}A_{\rm drive}/2 & \omega_{\rm s}^{(2)} - 2\omega_{\rm drive} & \cdots & 0 \\ \vdots & \vdots & \vdots & \ddots & \vdots \\ 0 & 0 & 0 & \cdots & \omega_{\rm s}^{(d-1)} - (d-1)\omega_{\rm drive} \end{pmatrix} + \begin{pmatrix} 0 & 0 & 0 & \cdots & 0 \\ 0 & B^{(1)}(t) & 0 & \cdots & 0 \\ 0 & 0 & B^{(2)}(t) & \cdots & 0 \\ \vdots & \vdots & \vdots & \ddots & \vdots \\ 0 & 0 & 0 & \cdots & B^{(d-1)}(t) \end{pmatrix}\right] \\
&\equiv \tilde{H}_{\rm S,RWA} + \hbar\sum_{j=1}^{d-1} B^{(j)}(t)|j\rangle\langle j|, \qquad (9)
\end{aligned}
$$

where we have introduced the system Hamiltonian $\tilde{H}_{\rm S,RWA} \equiv \hbar\sum_{j=1}\left[\left(\omega_{\rm s}^{(j)} - j\omega_{\rm drive}\right) + \frac{1}{2}\lambda^{(j-1,j)}A_{\rm drive}\left(\sigma_+^{(j-1,j)} + \sigma_-^{(j-1,j)}\right)\right]$, which does not include the dephasing term.

Next, we find a change-of-basis matrix V that diagonalizes the system Hamiltonian $\tilde{H}_{\rm S,RWA}$,

$$
\begin{aligned}
V^\dagger \tilde{H}_{\rm S,RWA} V =& \hbar V^\dagger \begin{pmatrix} 0 & A_{\rm drive}/2 & 0 & \cdots & 0 \\ A_{\rm drive}/2 & \omega_{\rm s}^{(1)} - \omega_{\rm drive} & \lambda^{(1,2)}A_{\rm drive}/2 & \cdots & 0 \\ 0 & \lambda^{(1,2)}A_{\rm drive}/2 & \omega_{\rm s}^{(2)} - 2\omega_{\rm drive} & \cdots & 0 \\ \vdots & \vdots & \vdots & \ddots & \vdots \\ 0 & 0 & 0 & \cdots & \omega_{\rm s}^{(d-1)} - (d-1)\omega_{\rm drive} \end{pmatrix} V \\
=& \hbar \begin{pmatrix} E^{(0)} & 0 & 0 & \cdots & 0 \\ 0 & E^{(1)} & 0 & \cdots & 0 \\ 0 & 0 & E^{(2)} & \cdots & 0 \\ \vdots & \vdots & \vdots & \ddots & \vdots \\ 0 & 0 & 0 & \cdots & E^{(d-1)} \end{pmatrix}, \qquad (10)
\end{aligned}
$$

where the eigenenergies $\hbar E^{(j)}$ of the j-th dressed states lie on its diagonal entries. Note that the matrix V may not diagonalize the full Hamiltonian $\tilde{H}_{\rm RWA}$. The frame where the system Hamiltonian $\tilde{H}_{\rm S,RWA}$ is diagnoalized is referred to as the spin locking frame. The full Hamiltonian in the spin locking frame is

$$
\begin{aligned}
\tilde{H}_{\rm SL} =& V^\dagger \tilde{H}_{\rm RWA}(t) V = V^\dagger \tilde{H}_{\rm S,RWA} V + V^\dagger\left(\hbar\sum_{j=1}^{d-1} B^{(j)}(t)|j\rangle\langle j|\right) V \\
=& \hbar\left[\mathrm{diag}(E^{(0)}, E^{(1)}, \cdots E^{(d-1)}) + \sum_{j=1}^{d-1} B^{(j)}(t) V^\dagger |j\rangle\langle j| V\right]. \qquad (11)
\end{aligned}
$$

Since the matrix V is not diagonal in general, the above equation clearly shows that longitudinal noise in the lab frame for a multi-level system leads to both transverse and longitudinal noise in the spin-locking frame. Note that this is not the case for a two-level system. In that case, longitudinal noise in the laboratory frame is fully transduced to transverse noise in the spin-locking frame.

For $\omega_{\rm drive} = \omega_{\rm s}^{(j)} - \omega_{\rm s}^{(j-1)}$, the relevant pair of the dressed states are $|+^{(j-1,j)}\rangle$, $|-^{(j-1,j)}\rangle$, with energy splitting $\hbar\Omega^{(j-1,j)} = (E^{(j)} - E^{(j-1)})$. This pair of dressed states spans an effective two-level subspace which forms the j-th spin-locking spectrometer. In order to describe the dynamics of the j-th spectrometer, we truncate the d-dimensional Hilbert space of the multi-level system to its two-dimensional subspace. The truncated Hamiltonian $\tilde{H}_{\rm SL}^{(j-1,j)}$ is given by

$$
\tilde{H}_{\rm SL}^{(j-1,j)} = \hbar\left[\begin{pmatrix} E^{(j-1)} & 0 \\ 0 & E^{(j)} \end{pmatrix} + \begin{pmatrix} \sum_{k=1}^{d-1} B^{(k)}(t)\langle j-1|V^\dagger|k\rangle\langle k|V|j-1\rangle & \sum_{k=1}^{d-1} B^{(k)}(t)\langle j-1|V^\dagger|k\rangle\langle k|V|j\rangle \\ \sum_{k=1}^{d-1} B^{(k)}(t)\langle j-1|V^\dagger|k\rangle\langle k|V|j\rangle & \sum_{k=1}^{d-1} B^{(k)}(t)\langle j|V^\dagger|k\rangle\langle k|V|j\rangle \end{pmatrix}\right]. \qquad (12)
$$

Note that the behavior of the j-th spectrometer is accurately described by Eq. (12) only if leakage out of this subspace can be neglected.

We evaluate the leakage rate from $|+\rangle^{(j-1,j)}$ (with eigenenergy $E^{(j)}$) to the energetically nearest dressed state outside of the subspace (with eigenenergy $E^{(j+1)}$) by applying Fermi's golden rule [S11], and analogously for $|-\rangle^{(j-1,j)}$. Note that here we consider leakage caused by dephasing noise, characterized by $B(t)$. The corresponding transition (leakage) rate $\Gamma_{\rm leak}^{(j)}$ can be expressed in terms of the noise spectral density $S(\omega) \equiv \int_{-\infty}^{\infty} \tau E^{-i\omega\tau}\langle B(\tau)B(0)\rangle$ as follows:

$$
\Gamma_{\rm leak}^{(j)} = \frac{A^2}{\hbar^2} S(\omega = E^{(j+1)} - E^{(j)}) \qquad (13)
$$

$A = \sum_{k=1}^{d-1} \langle j+1|V^\dagger|k\rangle\langle k|V|j\rangle$ denotes the matrix element for the corresponding transition. In the limit of a weak spin-locking drive as used in our experiment, the matrix element quantifying leakage out of the subspace is negligible compared to the matrix element between the spin-locked states, such that leakage is suppressed. In addition, the transition frequency to levels outside the spectrometer subspace is larger than the frequency splitting $\Omega^{(j-1,j)}$. For a noise PSD that decays for higher frequencies (e.g. $1/f$ noise), these high frequency transitions are less likely to be triggered. This effect additionally suppresses leakage.

Having validated the truncated Hamiltonian for our analysis, we re-express Eq. (S12) in terms of the Pauli operators for simplicity

$$\tilde{H}_{\mathrm{SL}}^{(j-1,j)} = \frac{\hbar}{2}\Omega^{(j-1,j)}\tilde{\sigma}_z^{(j-1,j)} + \hbar\left[\tilde{B}_\perp^{(j-1,j)}(t)(\tilde{\sigma}_+^{(j-1,j)} + \tilde{\sigma}_-^{(j-1,j)}) + \tilde{B}_\parallel^{(j-1,j)}(t)\left(\frac{\tilde{\sigma}_z^{(j-1,j)}}{2}\right)\right], \tag{14}$$

where $\tilde{\sigma}_z^{(j-1,j)}$, $\tilde{\sigma}_+^{(j-1,j)}$, and $\tilde{\sigma}_-^{(j-1,j)}$ denote the Pauli Z, raising, and lowering operators of the j-th spectrometer, respectively. We define the noise operators $\tilde{B}_\perp^{(j-1,j)}(t)$, $\tilde{B}_\parallel^{(j-1,j)}(t)$, describing longitudinal and transverse relaxation in the j-th spin locking subspace, respectively. They are given as linear combinations of the noise operators $B^{(j)}(t)$

$$\tilde{B}_\perp^{(j-1,j)}(t) = \sum_{k=1}^{d-1} B^{(k)}(t)\langle j-1|V^\dagger|k\rangle\langle k|V|j\rangle = \sum_{k=1}^{d-1} B^{(k)}(t)\langle j|V^\dagger|k\rangle\langle k|V|j-1\rangle \equiv \sum_{k=1}^{d-1}\alpha_{(j-1,j)}^{(k)}B^{(k)}(t), \tag{15}$$

$$\tilde{B}_\parallel^{(j-1,j)}(t) = \sum_{k=1}^{d-1} B^{(k)}(t)\Big[\langle j-1|V^\dagger|k\rangle\langle k|V|j-1\rangle - \langle j|V^\dagger|k\rangle\langle k|V|j\rangle\Big] \equiv \sum_{k=1}^{d-1}\beta_{(j-1,j)}^{(k)}B^{(k)}(t), \tag{16}$$

where the noise participation ratio $\alpha_{(j-1,j)}^{(k)}$ ($\beta_{(j-1,j)}^{(k)}$) is a dimensionless factor that quantifies the fraction of the energy fluctuation for level k that translates to the transverse (longitudinal) noise. The change-of-basis matrix V contains information about how the bare states mix and form the dressed states. By numerically estimating the matrix V, we calculated $\alpha_{(j-1,j)}^{(k)}$ as a function of A_{drive} (Fig. 3).

Supplementary Note 5 Derivation of Reduced Master Equation

We follow Ref. [S8] to derive a reduced master equation for the spin locking states of the j-th spectrometer. Note that the standard spin-locking theory discussed in Ref. [S8] does not include the longitudinal noise in the spin locking frame, from the multi-level nature of the sensor, as detailed above.

The starting point of this derivation is the Hamiltonian of the j-th spectrometer (Eq. (14)).

$$H_{\mathrm{SL}} = \frac{\hbar}{2}\Omega\sigma_z + \hbar\left[B_\perp(t)(\sigma_+ + \sigma_-) + B_\parallel(t)\left(\frac{\sigma_z}{2}\right)\right] \equiv H'_{\mathrm{S}} + H'_{\mathrm{SB}}, \tag{17}$$

Here, we have omitted the superscripts $(j-1,j)$ and the tildes for simplicity, and introduced $H'_{\mathrm{S}} \equiv \frac{\hbar}{2}\Omega\sigma_z$ and $H'_{\mathrm{SB}} \equiv \hbar\left[B_\perp(\sigma_+ + \sigma_-) + B_\parallel(\sigma_z/2)\right]$, which denote the spectrometer Hamiltonian and the spectrometer-bath interaction Hamiltonian respectively.

To employ the time convolutionless (TCL) projection operator technique [S8-S9], we move to the interaction picture with respect to the system Hamiltonian H'_{S}. Then, we have the interaction Hamiltonian H_{SB} as follows:

$$H_{\mathrm{SB}}(t) = \hbar\left[B_\perp(t)\left[e^{i\Omega t}\sigma_+ + e^{-i\Omega t}\sigma_-\right] + \frac{1}{2}B_\parallel(t)\sigma_z\right]. \tag{18}$$

To employ the technique, we assume the dephasing noise $B^{(j)}(t)$ is stationary noise with zero mean such that $\langle B^{(j)}(t)\rangle = 0$, $\langle B^{(j)}(t_1)\tilde{B}^{(j)}(t_2)\rangle = \langle B^{(j)}(t_1-t_2)\tilde{B}^{(j)}(0)\rangle$ and the coupling between the system and the bath is weak enough to truncate the time convolutionless generator at second order.

In this interaction picture, the equation of motion for the density matrix of the total system $\rho_{\mathrm{tot}}(t)$ is given as follows:

$$\frac{\partial}{\partial t}\rho_{\mathrm{tot}}(t) = -\frac{i}{\hbar}[H_{\mathrm{SB}}(t), \rho_{\mathrm{tot}}(t)] \equiv \mathcal{L}(t)\rho_{\mathrm{tot}}(t). \tag{19}$$

The Liouville super-opeartor $\mathcal{L}(t)$ has been defined as $\mathcal{L}(t)\cdot \equiv -i/\hbar[H_{\mathrm{SB}}(t), \cdot]$.

To apply the TCL technique, we assume the initial state of the total system is separable such that $\rho_{\mathrm{tot}}(0) = \rho_{\mathrm{S}}(0)\otimes\rho_B(0)$, where $\rho_S(t)$ and $\rho_B(t)$ denote the density matrix of the spectrometer and the bath in the interaction picture at time t, respectively. Now we introduce the projection superoperator

$$\mathcal{P}\cdot \equiv \rho_{\mathrm{B}}(0)\mathrm{Tr}_{\mathrm{B}}[\cdot], \tag{20}$$

which projects on the sensor part of the density matrix ρ_{tot}, where Tr_{B} denotes the tracing out operation over the bath. A complementary projection superoperator $\mathcal{Q}$ is then defined by $\mathcal{Q} \equiv I - \mathcal{P}$, where I is the identity superoperator. Using the above projection operators, we can write down the TCL master equation truncated at second order as follows:

$$\frac{\partial}{\partial t}\mathcal{P}\rho_{\text{tot}}(t) = \mathcal{K}(t)\mathcal{P}\rho_{\text{tot}}(t), \tag{21}$$

where $\mathcal{K}(t)$ denotes the second-order TCL generator and is given as

$$\mathcal{K}(t) = \int_0^t s\mathcal{P}\mathcal{L}(t)\mathcal{Q}\mathcal{L}(s)\rho_{\text{tot}}(t). \tag{22}$$

For noise with vanishing mean ($\langle\tilde{B}_\perp(t)\rangle = \langle\tilde{B}_\parallel(t)\rangle = 0$), substituting Eq. (18) into Eq. (21) and tracing over the bath leads to the following equation

$$\begin{aligned}\frac{\partial}{\partial t}\rho(t) = \int_0^t s\Bigg[&C_\perp(t,s)\left[e^{i\Omega(s-t)}\left[\sigma_+\rho(t)\sigma_- - \sigma_-\,\sigma_+\rho(t)\right] + e^{i\Omega(t-s)}\left[\sigma_-\rho(t)\sigma_+ - \sigma_+\,\sigma_-\rho(t)\right]\right]\\ &+ C_\perp(s,t)\left[e^{i\Omega(s-t)}\left[\sigma_-\rho(t)\sigma_+ - \rho(t)\sigma_+\,\sigma_-\right] + e^{i\Omega(t-s)}\left[\sigma_+\rho(t)\sigma_- - \rho(t)\sigma_-\sigma_+\right]\right]\\ &+ \frac{1}{4}C_\parallel(t,s)\left[\sigma_z\rho(t)\sigma_z - \sigma_z\sigma_z\rho(t)\right] + \frac{1}{4}C_\parallel(s,t)\left[\sigma_z\rho(t)\sigma_z - \rho(t)\sigma_z\sigma_z\right]\Bigg],\end{aligned} \tag{23}$$

which describes the evolution of the reduced density matrix of the system, $\rho(t) = \text{Tr}_{\text{B}}\rho_{\text{tot}}(t)$, in terms of the correlation functions

$$C_\perp(t,s) = \text{Tr}_{\text{B}}\left[B_\perp(t)B_\perp(s)\rho_{\text{B}}\right], \qquad C_\parallel(t,s) = \text{Tr}_{\text{B}}\left[B_\parallel(t)B_\parallel(s)\rho_{\text{B}}\right]. \tag{24}$$

Since we assumed that the bath-induced noise is a stationary process ($C_\perp(t,s) = C_\perp(t-s)$, $C_\parallel(t,s) = C_\parallel(t-s)$), we can substitute $s \equiv t - t'$, and rewrite the equation in the frequency domain as follows:

$$\begin{aligned}\frac{\partial}{\partial t}\rho(t) = \int_0^t t'\frac{1}{2\pi}\int_{-\infty}^{\infty}\omega e^{i\omega t'}\Bigg[&S_\perp(\omega)\left[e^{-i\Omega t'}\left[\sigma_+\rho(t)\sigma_- - \sigma_-\sigma_+\rho(t)\right] + e^{i\Omega t'}\left[\sigma_-\rho(t)\sigma_+ - \sigma_+\sigma_-\rho(t)\right]\right]\\ &+ S_\perp(-\omega)\left[e^{-i\Omega t'}\left[\sigma_-\rho(t)\sigma_+ - \rho(t)\sigma_+\sigma_-\right] + e^{i\Omega t'}\left[\sigma_+\rho(t)\sigma_- - \rho(t)\sigma_-\sigma_+\right]\right]\\ &+ \frac{1}{4}S_\parallel(\omega)\left[\sigma_z\rho(t)\sigma_z - \sigma_z\sigma_z\rho(t)\right] + \frac{1}{4}S_\parallel(-\omega)\left[\sigma_z\rho(t)\sigma_z - \rho(t)\sigma_z\sigma_z\right]\Bigg],\end{aligned} \tag{25}$$

where we have introduced the power spectral densities of noise operators, respectively as follows:

$$S_\perp(\Omega) = \int_{-\infty}^{\infty}\tau e^{-i\Omega\tau}\langle B_\perp(\tau)B_\perp(0)\rangle, \tag{26}$$

$$S_\parallel(\Omega) = \int_{-\infty}^{\infty}\tau e^{-i\Omega\tau}\langle B_\parallel(\tau)B_\parallel(0)\rangle. \tag{27}$$

Following Ref. [S9], we rewrite the equation by introducing a filter function $F(\omega) \equiv \int_0^t e^{i\omega t'}t'$ for the free induction decay [S11].

$$\begin{aligned}\frac{\partial}{\partial t}\rho(t) = \frac{1}{2\pi}\int_{-\infty}^{\infty}\omega\Bigg[&S_\perp(\omega)\Big[F(\omega-\Omega)\left[\sigma_+\rho(t)\sigma_- - \sigma_-\sigma_+\rho(t)\right] + F(\omega+\Omega)\left[\sigma_-\rho(t)\sigma_+ - \sigma_+\sigma_-\rho(t)\right]\Big]\\ &+ S_\perp(-\omega)\Big[F(\omega-\Omega)\left[\sigma_-\rho(t)\sigma_+ - \rho(t)\sigma_+\sigma_-\right] + F(\omega+\Omega)\left[\sigma_+\rho(t)\sigma_- - \rho(t)\sigma_-\sigma_+\right]\Big]\\ &+ \frac{1}{4}S_\parallel(\omega)F(\omega)\left[\sigma_z\rho(t)\sigma_z - \sigma_z\sigma_z\rho(t)\right] + \frac{1}{4}S_\parallel(-\omega)F(\omega)\left[\sigma_z\rho(t)\sigma_z - \rho(t)\sigma_z\sigma_z\right]\Bigg]\end{aligned} \tag{28}$$

The filter function $F(\omega)$ acts as a bandpass filter for the noise spectra, which is peaked at $\omega = 0$ with $1/t$ bandwidth. Assuming that all spectra vary negligibly over this passband, we approximate $F(\omega)$ by the delta function as follows:

$$\lim_{t\to\infty} F(\omega) = \pi\delta(\omega). \tag{29}$$

Then, the equation (S28) can be written as:

$$\begin{aligned}\frac{\partial}{\partial t}\rho(t) =& S_\perp(\Omega)\Big[\sigma_+\rho(t)\sigma_- - \frac{1}{2}\left[\sigma_-\sigma_+\rho(t) + \rho(t)\sigma_-\sigma_+\right]\Big]\\ &+ S_\perp(-\Omega)\Big[\sigma_-\rho(t)\sigma_+ - \frac{1}{2}\left[\sigma_+\sigma_-\rho(t) + \rho(t)\sigma_+\sigma_-\right]\Big]\\ &+ \frac{1}{4}S_\parallel(0)\left[\sigma_z\rho(t)\sigma_z - \frac{1}{2}\left[\sigma_z\sigma_z\rho(t) + \rho(t)\sigma_z\sigma_z\right]\right]\end{aligned} \tag{30}$$

From this equation, we find that the longitudinal decay rate $\Gamma_{1\rho}^{(j-1,j)}$ and the equilibrium-state polarization $\langle\tilde{\sigma}_z(t)\rangle|_{t\to\infty}$ in the spin-locking frame will be given as follows (we put the superscripts $(j-1,j)$ and tildes back for the sake of consistency with the main text):

$$\Gamma_{1\rho}^{(j-1,j)} = \tilde{S}_\perp(\Omega^{(j-1,j)}) + \tilde{S}_\perp(-\Omega^{(j-1,j)}) \tag{31}$$

$$\langle\tilde{\sigma}_z(t)\rangle|_{t\to\infty} = \frac{\tilde{S}_\perp(\Omega^{(j-1,j)}) - \tilde{S}_\perp(-\Omega^{(j-1,j)})}{\tilde{S}_\perp(\Omega^{(j-1,j)}) + \tilde{S}_\perp(-\Omega^{(j-1,j)})} \tag{32}$$

This result shows that transverse relaxation rate in the multi-level spin-locking experiment can be used to determine the noise spectral density of the longitudinal noise in the laboratory frame in the same way as for an ideal two-level system. Remarkably, the longitudinal noise PSD $\tilde{S}_\parallel$, which is an artifact of the multi energy level structure, does not enter Eqs. (S31, S32), which are consistent with the standard spin-locking analysis.

Supplementary Note 6 Contributions of T_1 decay to the spin relaxation $\Gamma_{1\rho}$

As discussed in Ref. [S12], energy relaxation (T_1 decay) of a two-level qubit sensor contributes to the longitudinal relaxation of the spin, locked at Rabi frequency Ω. This T_1 contribution to the spin relaxation can be expressed by the following equation:

$$\Gamma_{1\rho}(\Omega) = \frac{1}{2}\Gamma_1 + \Gamma_\varphi(\Omega) = \frac{1}{2}(\Gamma_{1\uparrow} + \Gamma_{1\downarrow}) + \Gamma_\varphi(\Omega). \tag{33}$$

Here $\Gamma_{1\rho}(\Omega)$ denotes the longitudinal spin relaxation rate, and $\Gamma_\varphi(\Omega)$ denotes the relaxation rate due to pure dephasing noise PSD at the locking Rabi frequency Ω. The energy relaxation rate $\Gamma_1 = 1/T_1$ of the qubit is given as a sum of an "up transition rate" $\Gamma_{1\uparrow}$ (from $|0\rangle$ to $|1\rangle$), and a "down transition rate" $\Gamma_{1\downarrow}$ ($|1\rangle$ to $|0\rangle$) [S13]. These up and down transition rates $\Gamma_{1\uparrow}$, $\Gamma_{1\downarrow}$ are determined by the noise spectrum $S_x(\omega)$ causing the energy relaxation at the qubit frequency ω_q [S8],

$$\Gamma_{1\uparrow} = S_x(-\omega_q) \text{ and } \Gamma_{1\downarrow} = S_x(+\omega_q). \tag{34}$$

Typically, qubits operate at low temperature and are assumed to be in thermal equilibrium with its cryogenic environment ($k_BT \ll \hbar\omega_q$). In this case, according to Maxwell-Boltzmann statistics, the up-rate $\Gamma_{1\uparrow}$ is exponentially smaller than the down-rate $\Gamma_{1\downarrow}$ by the Boltzmann factor $\Gamma_{1\uparrow}/\Gamma_{1\downarrow} = \exp[-(\hbar\omega_q)/(k_BT)]$. Therefore, in the low-temperature limit, we can approximate the relaxation rate Γ_1 as

$$\Gamma_1 \approx \Gamma_{1\downarrow} = S_x(+\omega_q). \tag{35}$$

Note that Eq. (33) relies on the assumption that the noise spectrum at qubit frequency $S_x(\pm\omega_q)$ varies negligibly within $\pm$ Rabi frequency $\pm\Omega$, such that $S_x(\omega_q) \simeq S_x(\omega_q \pm \Omega)$, and $S_x(-\omega_q) \simeq S_x(-\omega_q \pm \Omega)$ [S8].

Similarly, in the case of a multi-level sensor, we can apply Eq. (33) to the j-th spin-locked sensor in the weak driving limit (small $\Omega^{(j-1,j)}$),

$$\Gamma_{1\rho}^{(j-1,j)}(\Omega^{(j-1,j)}) = \frac{1}{2}\Gamma_1^{(j-1,j)} + \Gamma_\varphi(\Omega^{(j-1,j)}) = \frac{1}{2}\left(\Gamma_{1\uparrow}^{(j-1,j)} + \Gamma_{1\downarrow}^{(j-1,j)}\right) + \left(\tilde{S}_\perp(\Omega^{(j-1,j)}) + \tilde{S}_\perp(-\Omega^{(j-1,j)})\right) \quad , \tag{36}$$

where $\Gamma_1^{(j-1,j)} \equiv 1/T_1^{(j-1,j)}$ denotes the the energy relaxation rate between $|j-1\rangle$ and $|j\rangle$. Note that the above equation is valid only if the locking Rabi frequency, $\Omega^{(j-1,j)}$, is much smaller than the level anharmonicity of the sensor. If the $\Omega^{(j-1,j)}$ is comparable to level anharmonicity, then multi-level dressing effect must be taken into account.

To consider the multi-level dressing effect, we follow a similar approach to what we discussed in Supplementary Material 4. We estimate the matrix elements of the k-th raising and lowering operator, $\sigma_+^{(k-1,k)} \equiv |k\rangle\langle k-1|, \sigma_-^{(k-1,k)} \equiv |k-1\rangle\langle k|$, which are associated with the energy relaxation between $|k\rangle$ and $|k-1\rangle$, for the transition between the j-th spin-locked states: $\langle +^{(j-1,j)}|\sigma_\pm^{(k-1,k)}|-^{(j-1,j)}\rangle$ and $\langle -^{(j-1,j)}|\sigma_\pm^{(k-1,k)}|+^{(j-1,j)}\rangle$. Notably, these matrix elements can be estimated by using the change-of-basis matrix V, introduced in Supplementary Material 4. For instance, $\langle +^{(j-1,j)}|\sigma_+^{(k-1,k)}|-^{(j-1,j)}\rangle$ can be estimated as follows:

$$\langle +^{(j-1,j)}|\sigma_+^{(k-1,k)}|-^{(j-1,j)}\rangle = \langle j|V^\dagger\left(|k\rangle\langle k-1|\right)V|j-1\rangle. \tag{37}$$

Remarkably, the matrix elements for the transition between the j-th spin-locked states tell us at what rate the raising and lowering operator $\sigma_\pm^{(k-1,k)}$ (which are associated with T_1) will cause transitions between the spin-locked states. Namely, we can estimate the effective longitudinal spin relaxation rate, $\Gamma_{1,\text{eff}}^{(j-1,j)}$, as a consequence of the energy relaxation of a multi-level qubit, as

follows:

$$\begin{aligned}
\Gamma_{1,\text{eff}}^{(j-1,j)} &= \sum_k \langle |j-1|V^\dagger \left(\Gamma_{1\downarrow}^{(k-1,k)}|k-1\rangle\langle k| + \Gamma_{1\uparrow}^{(k-1,k)}|k\rangle\langle k-1|\right) V|j\rangle \\
&\quad + \langle |j|V^\dagger \left(\Gamma_{1\downarrow}^{(k-1,k)}|k-1\rangle\langle k| + \Gamma_{1\uparrow}^{(k-1,k)}|k\rangle\langle k-1|\right) V|j-1\rangle \\
&= \sum_k \langle |j-1|V^\dagger \left(S_x(+\omega_s^{(k-1,k)})|k-1\rangle\langle k| + S_x(-\omega_s^{(k-1,k)})|k\rangle\langle k-1|\right) V|j\rangle \\
&\quad + \langle |j|V^\dagger \left(S_x(+\omega_s^{(k-1,k)})|k-1\rangle\langle k| + S_x(-\omega_s^{(k-1,k)})|k\rangle\langle k-1|\right) V|j-1\rangle. \qquad (38)
\end{aligned}$$

Assuming the low effective temperature for the multi-level sensor ($\hbar\omega_s^{(k-1,k)} \gg k_B T$, we can replace $\Gamma_{1\downarrow}^{(k-1,k)} = S_x(+\omega_s^{(k-1,k)}) \approx \Gamma_1^{(k-1,k)}$, and $\Gamma_{1\uparrow}^{(k-1,k)} = S_x(-\omega_s^{(k-1,k)}) \approx 0$. Under this approximation Eq. (39) can be rewritten as:

$$\Gamma_{1,\text{eff}}^{(j-1,j)} \approx \sum_k \Gamma_1^{(k-1,k)} \left(\langle j-1|V^\dagger \left(|k-1\rangle\langle k|\right) V|j\rangle + \langle j|V^\dagger \left(|k-1\rangle\langle k|\right) V|j-1\rangle\right). \qquad (39)$$

Finally, the overall longitudinal relaxation rate $\Gamma_{1\rho}(\Omega)$ for a multi-level sensor, including the T1 decay contribution, is given as

$$\Gamma_{1\rho}^{(j-1,j)}(\Omega) = \frac{1}{2}\Gamma_{1,\text{eff}}^{(j-1,j)} + \Gamma_\varphi(\Omega). \qquad (40)$$

Note that if the energy relaxation rate is much faster than the dephasing rate at Rabi frequency $\Omega^{(j-1,j)}$ ($\Gamma_{1,\text{eff}}^{(j-1,j)} \gg \Gamma_\varphi(\Omega)$), the T_1 contribution can overshadow the contribution of dephasing noise. This T_1 overshadowing effect can make it hard to extract the dephasing noise, especially if T_1 is fluctuating. Namely, T_1 of the qubit sensor limits the noise sensitivity (= the smallest dephasing noise that can be detected reliably).

Supplementary Note 7 Separating out the contributions of T_1 decay and native dephasing

In the $\text{SL}^{(0,1)}$ and $\text{SL}^{(1,2)}$ experiments, we measure the longitudinal spin relaxation in both the presence and absence of the engineered noise to separate out the contributions of T_1 decay of the transmon sensor and native dephasing (T_φ) from the estimation of $\tilde{S}_\perp^{(j-1,j)}(\omega)$. The longitudinal spin relaxation rate in the presence of engineered noise for the j-th spin-locked spectrometer , $\Gamma_{1\rho}^{(j-1,j),\text{pres}}$ is given as

$$\Gamma_{1\rho}^{(j-1,j),\text{pres}} = \left(\tilde{S}_\perp^{(j-1,j)}(\Omega^{(j-1,j)}) + \tilde{S}_\perp(-\Omega^{(j-1,j)})\right) + \left(\tilde{S}_\perp^\varphi(\Omega^{(j-1,j)}) + \tilde{S}_\perp^\varphi(-\Omega^{(j-1,j)})\right) + \Gamma_1^{(j-1,j)}/2, \qquad (41)$$

where $\tilde{S}_\perp^\varphi(\Omega)$ corresponds to the power spectral density of the longitudinal spin relaxation noise, which is contributed from the native dephasing noise. The contribution of T_1 decay to the spin relaxation is denoted by $(\Gamma_1/2)$. Note that, for small Ω compared to the anharmonicity of the sensor, $\Gamma_1^{(j-1,j)}$ can be approximated as $1/T_1^{(j-1,j)}$, where $T_1^{(j-1,j)}$ is the lab-frame longitudinal relaxation time of the sensor for $|j-1\rangle$–$|j\rangle$ transition. As Ω increases, the peripheral states (neither $|j-1\rangle$ nor $|j\rangle$) start to participate to form the spin-locked states $\{|+\rangle^{(j-1,j)}, |-\rangle^{(j-1,j)}\}$. Hence, the longitudinal relaxation for the peripheral level transitions also start to contribute to $\Gamma_1^{(j-1,j)}$ as $\Omega^{(j-1,j)}$ increases. This contribution can be numerically accounted for by considering multi-level dressing as discussed in Sec. Supplementary Note 4.

The spin-polarization at equilibrium in the presence of engineered noise $\langle\sigma_z^{(j-1,j)}(t)\rangle|_{t\to\infty}^{\text{pres}}$ is given by

$$\langle\tilde{\sigma}_z^{(j-1,j)}(t)\rangle|_{t\to\infty}^{\text{pres}} = \frac{\tilde{S}_\perp(\Omega^{(j-1,j)}) - \tilde{S}_\perp(-\Omega^{(j-1,j)}) + \tilde{S}_\perp^\varphi(\Omega^{(j-1,j)}) - \tilde{S}_\perp^\varphi(-\Omega^{(j-1,j)})}{\tilde{S}_\perp(\Omega^{(j-1,j)}) + \tilde{S}_\perp(-\Omega^{(j-1,j)}) + \tilde{S}_\perp^\varphi(\Omega^{(j-1,j)}) + \tilde{S}_\perp^\varphi(-\Omega^{(j-1,j)})}. \qquad (42)$$

Accordingly, in the absence of the engineered noise, the spin-locking relaxation rate ($\Gamma_{1\rho}^{(j-1,j),\text{abs}}$) and the equilibrium spin-polarization ($\langle\tilde{\sigma}_z^{(j-1,j)}(t)\rangle|_{t\to\infty}^{\text{abs}}$) are given as follows:

$$\Gamma_{1\rho}^{(j-1,j),\text{abs}} = \tilde{S}_\perp^\varphi(\Omega^{(j-1,j)}) + \tilde{S}_\perp^\varphi(-\Omega^{(j-1,j)}) + \Gamma_1^{(j-1,j)}/2, \qquad (43)$$

$$\langle\tilde{\sigma}_z^{(j-1,j)}(t)\rangle|_{t\to\infty}^{\text{abs}} = \frac{\tilde{S}_\perp^\varphi(\Omega^{(j-1,j)}) - \tilde{S}_\perp^\varphi(-\Omega^{(j-1,j)})}{\tilde{S}_\perp^\varphi(\Omega^{(j-1,j)}) + \tilde{S}_\perp^\varphi(-\Omega^{(j-1,j)})}. \qquad (44)$$

Assuming that the engineered dephasing noise is much stronger than the native dephasing noise $\big(\tilde{S}_{\perp}(\Omega^{(j-1,j)})+\tilde{S}_{\perp}(-\Omega^{(j-1,j)}) \gg \tilde{S}^{\varphi}_{\perp}(\Omega) + \tilde{S}^{\varphi}_{\perp}(-\Omega^{(j-1,j)})\big)$, the $\langle\tilde{\sigma}_z^{(j-1,j)}(t)\rangle|^{\text{pres}}_{t\to\infty}$ can be approximated as follows:

$$
\begin{aligned}
\langle\tilde{\sigma}_z^{(j-1,j)}(t)\rangle|^{\text{pres}}_{t\to\infty} &\approx \frac{\tilde{S}_{\perp}(\Omega^{(j-1,j)}) - \tilde{S}_{\perp}(-\Omega^{(j-1,j)}) + \tilde{S}^{\varphi}_{\perp}(\Omega^{(j-1,j)}) - \tilde{S}^{\varphi}_{\perp}(-\Omega^{(j-1,j)})}{\tilde{S}_{\perp}(\Omega^{(j-1,j)}) + \tilde{S}_{\perp}(-\Omega^{(j-1,j)})} \\
&\approx \frac{\tilde{S}_{\perp}(\Omega^{(j-1,j)}) - \tilde{S}_{\perp}(-\Omega^{(j-1,j)})}{\tilde{S}_{\perp}(\Omega^{(j-1,j)}) + \tilde{S}_{\perp}(-\Omega^{(j-1,j)})} + \frac{\tilde{S}^{\varphi}_{\perp}(\Omega^{(j-1,j)}) + \tilde{S}^{\varphi}_{\perp}(-\Omega^{(j-1,j)})}{\tilde{S}_{\perp}(\Omega^{(j-1,j)}) + \tilde{S}_{\perp}(-\Omega^{(j-1,j)})}\langle\tilde{\sigma}_z^{(j-1,j)}(t)\rangle|^{\text{abs}}_{t\to\infty} \\
&\approx \frac{\tilde{S}_{\perp}(\Omega^{(j-1,j)}) - \tilde{S}_{\perp}(-\Omega^{(j-1,j)})}{\tilde{S}_{\perp}(\Omega^{(j-1,j)}) + \tilde{S}_{\perp}(-\Omega^{(j-1,j)})}
\end{aligned} \tag{45}
$$

From Eqs. (41, 43, and 45), we can solve for the $\tilde{S}_{\perp}(\Omega^{(j-1,j)})$ as follows:

$$
\tilde{S}_{\perp}(\Omega^{(j-1,j)}) = \frac{(1 + \langle\tilde{\sigma}_z^{(j-1,j)}(t)\rangle|^{\text{pres}}_{t\to\infty})}{2}\left(\Gamma_{1\rho}^{(j-1,j),\text{pres}} - \Gamma_{1\rho}^{(j-1,j),\text{abs}}\right) \tag{46}
$$

In addition, T_1 decay of the transmon sensor (mostly for $|j-2\rangle$–$|j-1\rangle$ transition) also results in the leakage of the j-th spin-locked states from the j-th spin-locking subspace. We effectively separated out this T_1-induced leakage from the the estimation of $\tilde{S}_{\perp}(\Omega^{(j-1,j)})$ by measuring the normalized spin polarization $\langle\tilde{\sigma}_z^{(j-1,j)}(t)\rangle = \frac{\rho^{(j-1,j-1)}(t)-\rho^{(j,j)}(t)}{\rho^{(j-1,j-1)}(t)+\rho^{(j,j)}(t)}$. Namely, we compensated the spin polarization for leakage of the spin-locked states $|+\rangle^{(j-1,j)}$ and $|-\rangle^{(j-1,j)}$ by multiplying a factor of $1/\big(\rho^{(j-1,j-1)}(\tau) + \rho^{(j,j)}(\tau)\big)$ to the spin-polarization, $\big(\rho^{(j-1,j-1)}(t) - \rho^{(j,j)}(t)\big)$.

Supplementary Note 8 Advantages of the spin-locking QNS over the dynamic decoupling QNS

Dynamic decoupling (D.D.) noise spectroscopy during predominantly free evolution has been widely used to characterize dephasing noise in various qubit systems. However, even within a two-level approximation, there are advantages to using a spin-locking (driven evolution) approach to noise spectroscopy. We discuss these advantages here to justify why we focus on extending the spin-locking based multi-level QNS throughout this work.

First, dynamic decoupling noise spectroscopy uses multiple, ideally instantaneous control pulses, which are used to flip the qubit states in the time domain and thereby realize a desired filter function in the frequency domain. Although we often think of these as narrow filters—ideally delta functions—that can sample the noise at any particular frequency, in practice, the width of the filter is determined by both the number of pulses and the duration of the experiment. Narrow filters require large numbers of pulses, which are not instantaneous in practice and take up available free-evolution time, and therefore lead to longer experiments (fighting against T_1). In turn, high-frequency spectroscopy requires small time separation between pulses, yet high-fidelity pulses are not boxcars, but generally have Gaussian or cosine envelopes, requiring a minimum time between pulses to remain accurate. Taken together, this trade space can be somewhat contradictory and may make high-frequency noise spectroscopy with high precision a challenge. Spin locking does not have these issues, because it is essentially a single quasi-continuous drive.

In practice, the DD control pulses are generally imperfect [1, 2]. These imperfections in control pulses (= control errors) limit the spectral range of the sensor, as discussed in Ref. [3]. Namely, the spectral range is mainly limited by control imperfections, rather than physical constraints inherent in the system. In contrast, the spin-locking noise spectroscopy does not require fast-pulsed control (it is a single quasi-CW drive); therefore, it can characterize higher frequency noise than the D.D. based noise spectroscopy as shown in Ref. [4] (this is also related to first point above).

In addition, practical D.D filters have a rather broad bandwidth (compared with spin locking), and a deconvolution step is required to extract the noise about some frequency. Furthermore, in many common approaches like the CPMG sequence, the filter function has one predominant peak and smaller lobes away from this peak, which exacerbates the deconvolution problem (a notable exception is the use of Slepian pulses, but these still lead to relatively broad filters). In contrast, the spin-locking approach uses a single, continuous drive, and we simply monitor decoherence times within the driven qubit basis to extract noise at the Rabi frequency with relatively small filter bandwidth (limited by coherence). This approach is much simpler to implement and generally more precise.

Supplementary References

[S1] R. Barends, J. Kelly, A. Megrant, D. Sank, E. Jeffrey, Y. Chen, Y. Yin, B. Chiaro, J. Mutus, C. Neill, P. OMalley, P. Roushan, J. Wenner, T. C. White, A. N. Cleland, and J. M. Martinis. Coherent Josephson qubit Suitable for scalable quantum integrated circuits. *Phys. Rev. Lett.* **111**, 080502 (2013).

[S2] J. Koch, T. M. Yu, J. Gambetta, A. A. Houck, D. I. Schuster, J. Majer, A. Blais, M. H. Devoret, S. M. Girvin, and R. J. Schoelkopf. Charge-insensitive qubit design derived from the Cooper pair box. *Phys. Rev. A* **76**, 042319 (2007).

[S3] E. Jeffrey, D. Sank, J. Y. Mutus, T. C. White, J. Kelly, R. Barends, Y. Chen, Z. Chen, B. Chiaro, A. Dunsworth, A. Megrant, P. J. OMalley, C. Neill, P. Roushan, A. Vainsencher, J. Wenner, A. N. Cleland, and J. M. Martinis. Fast accurate state measurement with superconducting qubits. *Phys. Rev. Lett.* **112**, 190504 (2014).

[S4] E. A. Sete, J. M. Martinis, and A. N. Korotkov. Quantum theory of a bandpass Purcell filter for qubit readout. *Phys. Rev. A* **92**, 012325 (2015).

[S5] Y. Sung, F. Beaudoin, L. M. Norris, F. Yan, D. Kim, J. Y. Qiu, U.V. Lüpke, J. L. Yoder, T. P. Orlando, S. Gustavsson, L. Viola, and W. D. Oliver. Non-Gaussian noise spectroscopy with a superconducting qubit sensor. *Nature Communications* **10**, 3715 (2019).

[S6] C. Macklin, K. O'Brien, D. Hover, M. E. Schwartz, V. Bolkhovsky, X. Zhang, W. D. Oliver, I. Siddiqi. A near-quantum-limited Josephson travelling-wave parametric amplifier. *Science* **350**, 307 (2015).

[S7] J. M. Gambetta, F. Motzoi, S. T. Merkel, and F. K. Wilhelm. Analytic control methods for high-fidelity unitary operations in a weakly nonlinear oscillator. *Phys. Rev. A* **83**, 012308 (2011).

[S8] U. von Lüpke, F. Beaudoin, L. M. Norris, Y. Sung, R. Winik, J. Y. Qiu, M. Kjaergaard, D. Kim, J. Yoder, S. Gustavsson, L. Viola, and W. D. Oliver. Two-Qubit spectroscopy of spatiotemporally correlated quantum noise in superconducting qubits. *PRX Quantum* **1**, 010305 (2020).

[S9] H. P. Breuer and F. Petruccione, *The Theory of Open Quantum Systems* (Oxford University Press, Oxford, 2002).

[S10] In the main text, we moved to the interaction picture with respect to the free Hamiltonian of the bath H_B, and introduced the time-dependent noise operator $B^{(j)}(t) \equiv e^{iH_\mathrm{B}t/\hbar}B^{(j)}e^{-iH_\mathrm{B}t/\hbar}$.

[S11] A. A. Clerk, M. H. Devoret, S. M. Girvin, F. Marquardt, and R. J. Schoelkopf. Introduction to quantum noise, measurement, and amplification. *Reviews of Modern Physics*, **82** (2010).

[S12] F. Yan, S. Gustavsson, J. Bylander, X. Jin, F. Yoshihara, D. G. Cory, Y. Nakamura, T. P. Orlando, and W. D. Oliver. Rotating-frame relaxation as a noise spectrum analyser of a superconducting qubit undergoing driven evolution. *Nature Communications*, **4** 2337 (2013).

[S13] P. Krantz, M. Kjaergaard, F. Yan, T. P. Orlando, S. Gustavsson, and W. D. Oliver. A quantum engineer's guide to superconducting qubits. *Applied Physics Reviews* **6**, 9021318 (2019).
